# Insights into the Structure and Protein Composition of *Moorella thermoacetica* Spores Formed at Different Temperatures

**DOI:** 10.3390/ijms23010550

**Published:** 2022-01-04

**Authors:** Tiffany Malleck, Fatima Fekraoui, Isabelle Bornard, Céline Henry, Eloi Haudebourg, Stella Planchon, Véronique Broussolle

**Affiliations:** 1 INRAE, Avignon Université, UMR SQPOV, F-84000 Avignon, France; tiffany.malleck@outlook.fr; 2 Unité EMaiRIT’S, CTCPA, F-84911 Avignon, France; fatima.fekraoui@agrosupdijon.fr; 3 INRAE, Pathologie Végétale, F-84143 Avignon, France; isabelle.bornard@inrae.fr; 4 PAPPSO, Micalis Institute, INRAE, AgroParisTech, Université Paris-Saclay, F-78352 Jouy-en-Josas, France; celine.henry@inrae.fr (C.H.); eloi.haudebourg@gmail.com (E.H.)

**Keywords:** electron microscopy, in silico analysis, proteomics, coat, exosporium

## Abstract

The bacterium *Moorella thermoacetica* produces the most heat-resistant spores of any spoilage-causing microorganism known in the food industry. Previous work by our group revealed that the resistance of these spores to wet heat and biocides was lower when spores were produced at a lower temperature than the optimal temperature. Here, we used electron microcopy to characterize the ultrastructure of the coat of the spores formed at different sporulation temperatures; we found that spores produced at 55 °C mainly exhibited a lamellar inner coat tightly associated with a diffuse outer coat, while spores produced at 45 °C showed an inner and an outer coat separated by a less electron-dense zone. Moreover, misarranged coat structures were more frequently observed when spores were produced at the lower temperature. We then analyzed the proteome of the spores obtained at either 45 °C or 55 °C with respect to proteins putatively involved in the spore coat, exosporium, or in spore resistance. Some putative spore coat proteins, such as CotSA, were only identified in spores produced at 55 °C; other putative exosporium and coat proteins were significantly less abundant in spores produced at 45 °C. Altogether, our results suggest that sporulation temperature affects the structure and protein composition of *M. thermoacetica* spores.

## 1. Introduction

Endosporulation is an ancient mechanism, first appearing about 2 billion years ago, that allows Gram-positive bacteria to adapt to harsh environmental conditions. Bacterial spores are much more resistant than vegetative cells and are considered to be dormant forms, providing sporulated bacteria the ability to persist in the environment for several years [1].

The structure of a spore differs greatly from that of vegetative cells, with the chromosome in a dehydrated core, surrounded by several concentric layers: the inner forespore membrane, the germ cell wall, the cortex, the outer forespore membrane, the coat layers, and an outer layer structure, called the crust in *Bacillus subtilis* and the exosporium in other species of *Bacillus* and *Clostridium* [2]. This particular structure has been shown to provide the spores some ability to adhere to biotic and abiotic surfaces, as well as protection against various types of stress [1,2].

Dehydration of the spore core is a major factor in spores’ resistance to wet heat, which increases as the core water content decreases [3,4]. The cortex appears to play an important role in reducing the water content of the spore during spore formation, together with the accumulation of dipicolinic acid (DPA), a specific component of spores that complexes with Ca^2+^ late in sporulation and displaces core water [5,6]. The two spore maturation proteins SpmA and SpmB have been shown to play a major role in spore core dehydration, as spores lacking these proteins are characterized by higher core hydration [3,4]. Furthermore, the small acid-soluble proteins (SASPs) α/β, which saturate and stabilize spore DNA, contribute to spores’ resistance to UV-C and dry heat, as well as to genotoxic compounds such as nitrous acid and formaldehyde [7,8,9,10]. The external layers formed by the proteinaceous coat are associated with protection against lysozyme and oxidizing agents [11,12,13,14].

These spore resistance properties, as well as spore structure and composition, depend strongly on the conditions of sporulation. Sporulation temperature is known to alter spores’ resistance to various stress, such as wet heat and biocides [15,16,17,18,19,20,21], and can have significant effects, together with medium composition, on spore structure and composition, such as fatty acid content and protein content [15,16,17,22,23,24].

The resistance of bacterial spores to extreme conditions represents a particular challenge in the food industry, where they can be a major cause of food spoilage. *Moorella thermoacetica* is a significant problem in the canned food industry because its spores are the most heat resistant of any so far retrieved, with *D* values (decimal reduction times) up to 110 min at 121 °C [20,25]. Formerly known as *Clostridium thermoaceticum, M. thermoacetica* is a strictly anaerobic and thermophilic spore-forming bacterium [26]. Recently, we showed that *M. thermoacetica* spores produced at the lowest temperature at which growth is possible (i.e., 45 °C) were less heat resistant and less resistant to biocides than spores produced at the optimal growth temperature (i.e., 55 °C) [27].

To date, *M. thermoacetica* spore structure has been only poorly described and its spore protein composition remains unknown. Here, we aimed to determine if *M. thermoacetica* spores produced at two different temperatures, 45 °C and 55 °C, exhibited differences in terms of structure and protein composition. To achieve this, the surface structure and ultrastructure of spores produced at these two temperatures were observed by scanning electron microscopy (SEM) and transmission electron microscopy (TEM), respectively. Spore protein composition was then further analyzed using an LC-MS/MS label-free approach.

Using this approach, we are able to report that the coat ultrastructure of *M. thermoacetica* spores and the protein composition of spore layers are modified by suboptimal sporulation conditions.

## 2. Results

### 2.1. Spore Surface Structure

**Figure 1:**
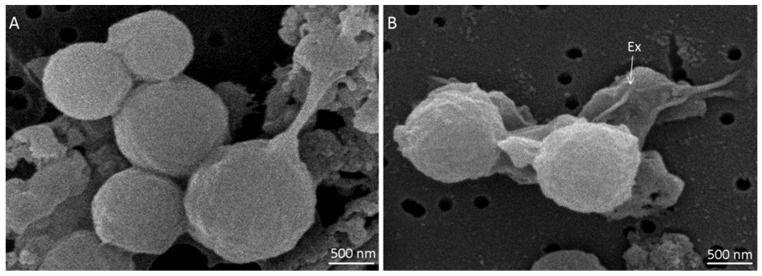
Spores of *Moorella thermoacetica* strain ATCC 39073, produced on agar plates at 55 °C and observed by scanning electron microscopy: (**A**) spores of different sizes with a smooth surface; (**B**) two spores enveloped by a large, loose structure corresponding to the exosporium (Ex), as indicated by the white arrow.

Spores of *M. thermoacetica* ATCC 39073 were produced on agar plates at either optimal growth temperature (55 °C) or suboptimal temperature (45 °C), with four batches of each. They were then observed by SEM (*n* = 78 and *n* = 73 spores, respectively). Spores produced at 55 °C appeared spherical and highly heterogeneous in terms of size (Figure 1A).

Indeed, spore diameter ranged from 0.87 to 1.65 µm, with an average size of 1.2 ± 0.1 µm. Spores produced at 45 °C exhibited the same morphology as at 55 °C (Figure S1), but were significantly smaller (*p* < 0.05), with a diameter ranging from 0.85 to 1.46 µm and an average size of 1.1 ± 0.1 µm (Tables S1 and S2). A large, loose structure was observed surrounding the spores (Figure 1B). This structure, which is assumed to be the exosporium, was more easily distinguished when spores were formed at 45 °C. The spore surface structure, as observed by SEM, did not seem to differ based on sporulation temperature.

### 2.2. Spore Ultrastructure

**Figure 2:**
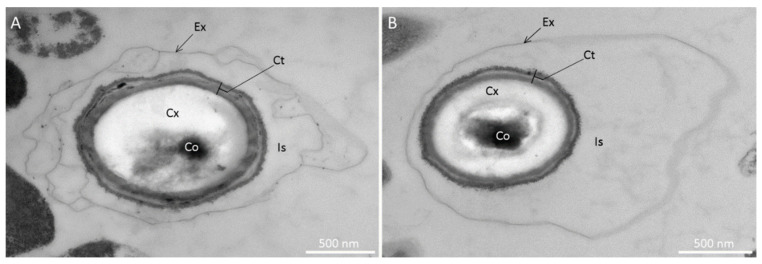
Ultrastructure of spores of *Moorella thermoacetica* ATCC 39073 produced at 55 °C (**A**) or 45 °C (**B**) on agar plates, observed by transmission electron microscopy. Ex: exosporium; Is: interspace; Ct: coat; Cx: cortex; Co: core.

To determine if the spore ultrastructure is affected by sporulation temperature, we used TEM to describe *M. thermoacetica* spores produced at 55 °C or 45 °C (four independent batches; *n* = 38 and *n* = 45 spores, respectively). Spores produced at 55 °C were characterized, from the inner to the outer structure, by the presence of a core, a cortex and a coat, all surrounded by a large, loose exosporium, which appeared to be separated from the coat by an interspace (Figure 2A). The exosporium was observed, regardless of the sporulation temperature. The global structure of spores produced at 45 °C was very similar (Figure 2B); however, the border between the core and the cortex layer was more easily observed than in spores produced at 55 °C (Figure 2A,B).

**Figure 3:**
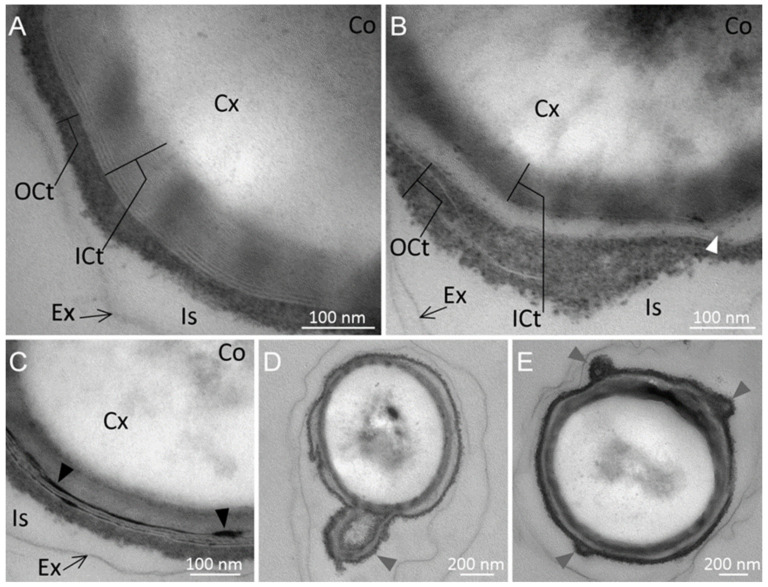
Coat structure of *Moorella thermoacetica* ATCC 39073 spores produced at 55 °C (panels (**A**,**C**) and at 45 °C (panels (**B**,**D**,**E**)). Two types of spores were obtained under both sporulation conditions (panels (**A**,**B**)). The spore coat in panel (**A**) is made of an inner lamellar layer in direct contact with a dense, diffuse outer layer, while in panel (**B**) the spore inner and outer coats are separated from each other by a less electron-dense zone (white arrow). Panel (**C**) shows electron-dense deposits often observed in the inner spore coat of spores produced at 55 °C (black arrows). Outer and inner spore coat misarrangements are shown in panels (**D**,**E**) (gray arrows). Ex: exosporium; Is: interspace; OCt: outercoat; ICt: innercoat; Cx: cortex; Co: core.

Overall, two types of spores were observed: (1) spores with a lamellar inner coat in direct contact with a thinner, electron-dense and diffuse outer coat layer (Figure 3A), mainly observed in spores produced at 55 °C (30 spores out of the 45 observed); and (2) spores exhibiting the same inner and outer coat layers, which seemed to be separated by a less electron-dense zone (Figure 3B), mainly observed in spores produced at 45 °C (34 spores out of the 39 observed). Moreover, we sometimes observed the presence of highly electron-dense material accumulated in the inner spore coat layer, either surrounding the spore or present as small deposits (Figure 3C).

These structures were more frequently observed in spores produced at 55 °C than at 45 °C (18 and 2 spores, respectively). Moreover, some spores exhibited misarranged coats, which were loosely attached to the spore surface at some points, with protuberances in the outer coat (Figure 3E) or the outer coat and part of the inner coat partially detached from the spore surface (Figure 3D). These misarrangements were more frequently observed in spores produced at 45 °C than at 55 °C (17 out of the 39 observed spores and 9 out of 45 spores, respectively).

### 2.3. Identification of Spore Proteins in Moorella thermoacetica ATCC 39073

To identify spore-associated proteins in the genome of *M. thermoacetica* ATCC 39073 [28], we performed a BLAST search using amino-acid sequences of spore proteins, identified in previous proteomic or genomic analysis on *Clostridium difficile*, *Clostridium perfringens*, *Bacillus subtilis*, *Bacillus cereus*, and *Bacillus anthracis* (see references in Table 1), belonging to the Clostridiales and Bacillales orders of the Firmicutes [29]. We mainly focused on proteins from the coat and exosporium layers, as well as proteins described as being involved in spore resistance properties. We searched for the presence/absence of about 200 spore-associated proteins (Table 1 and Table S2). From this, a total of 62 putative orthologs was identified in the *M. thermoacetica* genome (Table 1), with 51 of those corresponding to proteins known to be localized in the coat, exosporium, or spore membranes in other spore-forming bacteria.

Among the proteins associated with the exosporium, we identified orthologs of two rubrerythrins from *C. difficile*, as well as orthologs of CD0116, CD0117, and CD0118, which might play a role in oxidative stress resistance [30,31]. The *moth_2167* gene was found to encode a putative alanine racemase and two putative arginases were also identified. Among the proteins associated with the coat, the *moth_1319* gene appeared to encode an ortholog of the morphogenetic protein SpoIVA, which is necessary for proper assembly of the inner coat of *B. subtilis*. Among the structural coat proteins, we found orthologs of the inner coat layer proteins CotJB and CotJC, a putative CotSA ortholog, and two coat F-containing proteins. One of these latter proteins, encoded by the *moth_1782* gene, was identified according to genome annotation and a blastP search against *Clostridium* proteins confirmed that this protein likely corresponds to an unidentified coat protein. The second coat F-containing protein, encoded by the *moth_2016* gene, is homologous to the uncharacterized protein YhcQ of *B. subtilis*. In addition, we found an ortholog of a YabG protease that is associated with spore coat protein modification [32]. Among the spore coat proteins, we also identified a superoxide dismutase protein, which is thought to play a potential role in spore resistance to hydrogen peroxide [31]. Other putative spore coat proteins included the spore cortex-lytic hydrolases YaaH, YdhD, and YhxC, for which an additional role in *B. subtilis* competence has recently been reported [33]; and several proteins, such as YkvN, whose roles have not yet been described.

In this in silico analysis, we also identified proteins that are not described as coat or exosporium proteins but were isolated from spore coat and/or exosporium fractions in other spore formers. Among these, we found orthologs of proteins involved in germination such as Gpr, SleB, or YpeB, as well as an ortholog of the DacB protein, which modifies the degree of glycan cross-linking in the cortex [39]. Finally, we also identified six proteins corresponding to orthologs of α/β-SASPs, which have been implicated in spore resistance, as well as the proteins SpmA, SpmB, SpoVFA, and SpoVFB, which are involved in spore core hydration [4,7,8,10].

### 2.4. Proteomic Analysis of Spores Formed at Different Temperatures

A proteomic analysis was performed on total protein extracts of spores of *M. thermoacetica* ATCC 39073 produced at either 45 °C or 55 °C, which enabled the identification of a total of 1018 proteins (Tables S3 and S4). Of these, 91 were significantly differentially produced in spores formed at 45 °C versus 55 °C (Table S3).

Of the 62 putative spore-associated proteins identified by our in silico analysis, 24 were identified by LC-MS/MS and changes in their relative abundance between the two sporulation conditions were determined (Table 2). Based on localization data reported for other spore-forming bacteria, we found 7 putative exosporium-related proteins, 11 putative coat proteins, and 6 proteins that were putatively localized in the core, in the inner membrane, or had no assigned localization (Table 1 and Table 2).

Nineteen proteins were identified in both sets of spores (45 °C and 55 °C), although nine of these were significantly less abundant (padjust_value < 0.05) when spores were produced at 45 °C than at 55 °C (Table 2). For example, the exosporium oxidoreductase protein (Q2RIB2) was quantified with an average of 32 ± 4.6 versus 53 ± 4.6 spectra, respectively. Similar patterns were detected for the coatF-like protein Q2RHK6 (2.3 ± 2.1 versus 9.7 ± 3.2 spectra), the CotJC protein (1.7 ± 1.5 versus 8.3 ± 4.7 spectra), and the putative YmxG coat protein (2.3 ± 0.6 versus 8.3 ± 1.2 spectra in spores produced at 45 °C and 55 °C, respectively). The SpoIVA protein, which plays a role in spore coat formation, was detected with an average of 21.3 ± 12 versus 41 ± 6.6 spectra in spores produced at 45 °C and 55 °C, respectively.

Five proteins were identified in only one sporulation temperature condition (Table 2): the proteins CotSA, YpeB, SodF, the 2-oxoglutarate synthase (Q2RMG5), and CotJB were only identified in spores produced at 55 °C. Significant differences in relative abundance were noted for CotSA, YpeB, and SodF (padjust_value = 0.0002, 0.0057 and 0.0275, respectively), while CotJB and Q2RMG5 were present only at low levels in spores produced at 55 °C, and only in one and two of the three biological replicates, respectively (Table 2). We also noticed a high variability in the relative abundance of certain proteins, such as YpeB, SodF, and the oxidoreductase YqiG, which was due to a low number of spectral counts for these proteins and the variability among replicates of protein extracts. The potential exosporium-related arginase (Q2RHH4) was detected with 2.7 ± 1.2 spectra in spores produced at 45 °C and 1.3 ± 1.2 spectra in spores produced at 55 °C.

## 3. Discussion

### 3.1. Coat Layers of Moorella thermoacetica Differ According to Sporulation Temperature

Previous work by our group revealed that spores of *M. thermoacetica* ATCC 39073 that were produced on solid medium at 45 °C were less resistant to wet heat and peracetic acid (PAA) than spores produced at 55 °C [27]. Here, we investigated whether these differences in resistance could be explained by modification in the structure and/or protein composition of spores formed at different sporulation temperatures.

Using SEM and TEM, we characterized the spore surface and ultrastructure of *M. thermoacetica* spores formed at either 45 °C or 55 °C. To our knowledge, this work produced the first SEM images describing spore surface features of *M. thermoacetica*. These SEM observations showed that the spores were spherical and had a similar shape regardless of sporulation temperature. However, spores produced at the optimal temperature (55 °C) were significantly bigger than spores produced at 45 °C, as previously reported for *B. weihenstephanensis* [18]. At both sporulation temperatures, we observed a loose structure on the spore surface, which we assumed to be the exosporium layer.

Observations using TEM revealed that *M. thermoacetica* spores exhibited the layers classically described in bacterial endospores, namely, the cortex, the coat, and a large exosporium surrounding the spore. The presence of an exosporium was previously described for spores of *M. thermoacetica* JW/DB-2 [20]; in this strain, as for *C. difficile*, the exosporium is in close contact with the spore coat [20,36]. In contrast, the exosporium of *M. thermoacetica* ATCC 39073 seemed to be loose and separated from the coat layers by an interspace, as observed in *B. cereus* and *B. anthracis* [40]. Moreover, the exosporium surface of *Moorella* spores lacked any discernable “hair-like” projections, which have been reported in spores of *C. difficile*, *C. sporogenes*, *B. cereus*, and *B. anthracis* [36,37,41,42,43].

While the structure of the exosporium did not seem to differ between sporulation temperatures, we observed that the coat structure was indeed altered. Most of the spores produced at 55 °C exhibited a coat composed of a lamellar inner coat in direct contact with a thinner and more diffuse, but electron-dense, outer coat, while the inner and outer coat of spores produced at 45 °C were more frequently separated by a less electron-dense intermediate layer. A similar pattern was previously observed for *B. subtilis* spores produced at suboptimal temperature [44]. Moreover, spore coat misarrangements were more frequently observed for spores produced at 45 °C than at 55 °C. We hypothesize that the coat may be less organized when spores are produced at temperatures closer to the lower limit possible for growth. In line with this, we also noticed that the spore core and cortex were more easily observed in spores produced at 45 °C. This observation might be due to a better permeation of staining inside spores produced at the lower temperature and, thus, certain structural differences.

Taken together, these observations might help to explain the weaker resistance properties we previously reported for *M. thermoacetica* spores produced at 45 °C [27]. In a similar way, previous research has reported that *B. subtilis cotE* spores that lack most coat proteins were more sensitive to glutaraldehyde, lysozyme, and oxidizing agents [11,12,13,14]. Another study showed that *B. cereus* spores lacking CotE exhibited coat defects and were less resistant to lysozyme and an oxidizing agent; interestingly, though, the mutant spores were also more resistant to heat [45].

### 3.2. Identification of Spore Protein Orthologs in Moorella thermoacetica ATCC 39073

To further investigate the differences in coat structure, we analyzed and compared the protein composition of *M. thermoacetica* spores produced at 45 °C and 55 °C. We chose to focus our data analysis on proteins that have been linked with the spore coat and exosporium in other spore formers and on proteins associated with spore resistance properties.

As the *M. thermoacetica* ATCC 39073 genome is poorly annotated with respect to these proteins, we first searched for orthologs for about 200 proteins that have been previously retrieved from coat or exosporium fractions of other spore-forming bacteria. In total, about 25% of these had orthologs in *M. thermoacetica* [34,35,37,38]. None of the morphogenetic proteins that have been reported as components of the *B. cereus* or *B. anthracis* exosporium—such as ExsA, ExsY, CotY, BxpB, ExsM, and ExsFB [38,41,42,46]—were identified in the *M. thermoacetica* genome. We did identify putative orthologs of several *C. difficile* exosporium proteins, but not of the morphogenetic CdeC, CdeM, or BclA proteins (Table S2) [37,38]. As TEM images clearly showed the presence of a large exosporium surrounding the *M. thermoacetica* spores, we hypothesize that the *Moorella* exosporium proteins are different from those of other spore formers, including closely related *Clostridium* species. Indeed, spore surface proteins are thought to be subjected to a great deal of evolutionary pressure for adaptation to different ecological niches [41]. The in silico analysis also allowed us to identify an ortholog of the well-conserved inner coat morphogenetic protein, SpoIVA [47,48,49,50]. However, no matches were found for the morphogenetic coat proteins SafA and CotE, which are essential for the assembly of the coat basement layer and the outer coat layer, respectively, in *B. subtilis* and *B. cereus* spores [41,45]. This observation is consistent with the fact that these proteins are not conserved in Clostridia [41,47,48,50]. Furthermore, we found no equivalent in *M. thermoacetica* for the spore coat morphogenetic protein SipL, which plays an essential role in spore coat assembly in *C. difficile* by interacting with SpoIVA [51,52,53]. SipL is thought to be a homolog of the *B. subtilis* spore coat morphogenetic protein SpoVID, which does not seem to be conserved in Clostridia or *M. thermoacetica* [41,48,50]. Moreover, although it has been hypothesized that the *C. difficile* CotA protein could play a role in outer spore coat assembly, similarly to the *Bacillus* CotE protein [54], our in silico analysis did not identify any CotA ortholog in the *M. thermoacetica* genome.

Apart from spore coat morphogenetic proteins, few coat proteins were identified by the in silico analysis. Among the proteins known to be associated with the inner coat, we identified CotJB and CotJC, but not CotJA. In *B. subtilis*, these three proteins are encoded by the tricistronic operon *cotJA-cotJB-cotJC* [55]; however, a comparative genomic study showed that the CotJA, CotJB, and CotJC orthologs are not always found together [47]. Our analysis also identified a YhaX ortholog, corresponding to a spore-coat basement protein, as well as an ortholog of the *C. difficile* CotG catalase [54]. However, we found no ortholog of *B. subtilis* CotG, which has a negative effect on CotU, CotC and CotS assembly [56]; these three coat proteins were likewise absent in the *M. thermoacetica* genome.

Several orthologs of inner membrane proteins were present in *M. thermoacetica*, including three putative spore cortex lytic enzymes (SleB) and the YpeB protein, which has been shown to be required for SleB stabilization, via protein–protein interactions, and subsequent germination of *B. anthracis* spores [57]. We also identified orthologs of two other widely conserved germination proteins, Gpr and YtfJ/GerW [49]. Moreover, we found two serine-type D-Ala-D-Ala carboxypeptidases that correspond to putative orthologs of DacB (the first and second BlastP hits were Q2RIR9 (moth_1058) and Q2RJL7 (moth_1357), respectively), which is involved in peptidoglycan cross-linking [4,39,58]. In *B. subtilis*, the *dacB* gene is located in an operon with *spmA* and *spmB*, while in *C. perfringens dacB* is monocistronic [4]. In *M. thermoacetica*, SpmA and SpmB are encoded by *moth_1356* and *moth_1355*, respectively. As the Q2RIR9 and Q2RJL7 proteins were similar, we considered them both to be potential DacB proteins. Interestingly, we identified several orthologs encoding proteins that are thought to play a role in preventing spore germination, such as the alanine racemase Alr, or that have been implicated in spore resistance, such as the coat protein SodF, which is associated with a potential resistance to oxidizing agents, and two potential exosporium-related rubrerythrins. However, as mentioned above, the CotA protein, which is associated with resistance to oxidizing chemicals in *B. subtilis* but is absent in Clostridia [41], was not identified here.

We also identified orthologs for all the spore resistance-associated proteins localized in the inner spore layers, including the α/β-type SASPs SspA and SspF. Several studies have shown that α/β-type SASPs in Bacilli and Clostridia play a major role in protecting spore DNA against UV-C and genotoxic chemicals [1,8,10]. We also found orthologs of the two conserved proteins SpmA and SpmB, which play an essential role in spore core hydration [1,3,4,39,49], as well as SpoVFA and SpoVFB, which are essential for DPA production. DPA chelates with Ca^2+^ to form Ca-DPA, which is imported into the forespore core and lowers the spore water content, thus improving its resistance properties [2]. Ca-DPA has been shown to play a role in spore resistance to both heat and chemicals [5,31,59].

### 3.3. Proteins Extracted from Moorella thermoacetica ATCC 39073 Spores Differ Based on Sporulation Temperature

Our morphological analysis revealed that spore structure in *M. thermoacetica*, and more precisely, the arrangement of the coat layers, seemed to be modified by sporulation temperature at a temperature close to the lower limit for growth. We therefore investigated if the differences we observed in coat structure could be due to a difference in protein composition.

A proteomic analysis allowed the identification of 1018 spore proteins (Tables S3 and S4). However, despite the purification steps we carried out after spore harvesting, it is possible that spore suspensions may have contained some debris from vegetative cells, due to the low sporulation efficiency of this bacterium; indeed, an earlier report on another strain of *M. thermoacetica* found that only 5% of the cells formed spores [60]. For this reason, we focused only the putative spore structural or spore-associated proteins, identified in our in silico analysis.

Of the 62 spore proteins identified in the genome of *M. thermoacetica* ATCC 39073, 24 were detected by LC-MS/MS analysis. We found seven proteins that were potentially associated with the exosporium layer, such as the two rubrerythrins Rbr, encoded by the *moth_1286* and *moth_1279* loci [31], as well as a putative arginase [61], an enolase, and an alanine racemase. The protein product of the *moth_1518* locus, which was identified as an ortholog of an uncharacterized exosporium protein, was significantly less abundant in spores produced at 45 °C, while the putative arginase encoded by the *moth_1815* locus was slightly more abundant.

Among the 11 putative coat proteins retrieved, the coat morphogenetic protein SpoIVA appeared to be less abundant in protein extracts from spores produced at 45 °C than at 55 °C (padjust_value < 0.05). Given that our TEM analysis found that spores produced at 45 °C exhibited more coat misarrangement, and that SpoIVA is essential for coat formation [41], it is tempting to hypothesize that the lower amount of this protein in spores produced at 45 °C may lead to a reduced cohesion of the *M. thermoacetica* spore coat.

Four spore coat proteins were less abundant in protein extracts of spores produced at the suboptimal compared to the optimal temperature, such as the coat-F containing protein, encoded by the *moth_1782* locus and the inner coat protein CotJC. Instead, CotSA and SodF were not detected in spores produced at 45 °C. In *C. difficile*, the superoxide dismutase SodF likely plays a role in polymerizing the spore coat protein monomers by oxidative cross-linking in the presence of H_2_O_2_, while CotJC might be involved in resistance to oxidative stress, although in both cases their protective role remains to be elucidated [41,54]. If SodF and CotJC do in fact play a protective role and are less abundant in spores produced at 45 °C than at 55 °C, this might explain, at least partially, the lower resistance of *M. thermoacetica* spores to peracetic acid when formed at a suboptimal temperature [27].

Altogether, we report that certain proteins were relatively less abundant in spores produced at 45 °C than in those formed at 55 °C. This could be due to a true difference in abundance between spores formed at different temperatures and/or that a different structuration of spores allowed these proteins to be more readily extracted at 55 °C. Our results do not allow us to determine which hypothesis is valid, but overall, they point to modifications in *M. thermoacetica* spore coat composition and/or structure that appear to be based on sporulation temperature. In this, our study is in agreement with work in *B. subtilis*, showing that the amount of extracted outer spore coat proteins, such as CotA, CotU, CotG, or CotQ, as well as crust proteins, such as CotX, CotY, and CotZ, varied depending on the sporulation conditions [23]. It was also shown that fewer CotA and CotS proteins were extracted from *B. subtilis* spores produced at a high temperature [15], while another recent study revealed that CotG and CotB were more abundantly extracted from spores produced at 25 °C than from spores produced at 42 °C [44].

In conclusion, we investigated the structure and protein composition of *M. thermoacetica* spores produced at optimal and low-limit growth temperatures. Using electron microscopy, we showed that spores produced at 45 °C were smaller than those formed at 55 °C. Moreover, spores exhibited some differences in the inner coat structure depending on the sporulation temperature. We found that the spore protein composition varied between sporulation temperatures and, more precisely, that fewer spore coat proteins were extracted from spores produced at 45 °C. It is possible to hypothesize that the morphological differences observed between spores produced at 45 °C and 55 °C could be due to (1) a lower abundance of structural proteins in spores produced at 45 °C, leading to a more loosely arranged coat aspect; and/or (2) differences in protein cross-linking between spores produced at 45 °C and 55 °C. The differences observed in terms of protein composition and, in particular, the lower abundance of SodF and certain structural coat proteins in spores produced at 45 °C might be involved in the differing resistance properties of these spores to wet heat and biocides. However, more work is needed to improve the purification process for *Moorella* spores, as well as the extraction of insoluble spore proteins to (1) provide a more thorough characterization of *M. thermoacetica* spore proteins and those associated with specific layers (i.e., coat and exosporium); and (2) confirm the differences we noticed in spore protein composition between the two temperature conditions and highlight other potential variations. Indeed, several parameters play a role in spore resistance to chemicals and wet heat, such as DPA content, spore core dehydration, and inner-membrane fatty-acid content [16,22,62]. It would therefore be interesting to further characterize *M. thermoacetica* spores in order to better understand how sporulation temperature affects *Moorella* spore resistance, to decipher how these spores are able to exhibit such robust resistance properties.

## 4. Material and Methods

### 4.1. Strain and Spore Production

This study was performed with the *Moorella thermoacetica* ATCC 39073 strain. The bacteria were stored in a cryogenic preservative solution with beads (AES Chemunex, Bruz, France). Pre-cultures were performed by dropping three beads in 10 mL of modified deoxygenated DTB medium (_mod_DTB: 9 g/L tryptone, 4 g/L tryptose, 7 g/L soytone, 5 g/L yeast extract, 5 g/L sodium chloride, 1 g/L potato starch, and 10 g/L D-glucose). All components were purchased from Biokar Diagnostics, (Allonne, France) or Thermo Fisher Diagnostics (Dardilly, France). Pre-cultures were incubated at 55 °C for 4 to 6 days in anaerobiosis with paraffin. A volume of 200 µL of pre-culture was inoculated into 10-mL tubes of _mod_DTB that have been previously reduced in an anaerobic chamber for 24 to 48 h (90% N_2_, 5% H_2_, 5% CO_2_, Whitley A35 Workstation, Don Whitley Scientific, Bingley, UK). Anaerobiosis was maintained with paraffin and cultures were incubated either at 55 °C for 4 to 5 days or at 45 °C for 13 days. Sporulation was then promoted by pouring 2 mL of cultures onto the surface of 140-mm-diameter meat liver agar plates (MLA, Biokar Diagnostics, Allonne, France). Plates were incubated under anaerobiosis (Oxoïd™ AnaeroGen™ jars and sachets, Sigma-Aldrich, Saint-Quentin-Fallavier, France) at either 45 °C or 55 °C. Sporulation was checked and stopped after 3 to 4 weeks of incubation; plates were incubated at room temperature for an additional week. Spores were then harvested with cold sterile water, centrifuged at 1400× *g* for 20 min at 4 °C, and resuspended in sterile water. Unless otherwise stated, the suspensions were heat-treated at 100 °C for 10 min to inactivate the residual vegetative forms and germinated spores [27]. Spore suspensions were stored at 4 °C until use.

### 4.2. Spore Purification

One-milliliter aliquots of heat-treated spore suspensions were briefly centrifuged at 200× *g* for 1 min at 4 °C and the supernatant was removed. The spore purification protocol, adapted from Aoyama [60], was as follows: spores were resuspended in 1 mL of 10 mM Tris-HCl buffer pH 8.0 containing 1.7 µg/µL lysozyme (Sigma Aldrich, Saint-Quentin Fallavier, France) and incubated overnight at 37 °C with continuous stirring. Lysis of remaining vegetative cells was checked under a phase-contrast microscope (Leica DM750, France). Spore suspensions were washed three times in 1 mL of cold sterile distilled water, centrifuged at 5000× *g* for 10 min at 8 °C, resuspended in 1 mL of 1% SDS (*v*/*v*) (Sigma Aldrich, Saint-Quentin Fallavier, France), and incubated at 37 °C for 2 h with continuous stirring. Spores were then washed three times in cold sterile distilled water at 5000× *g* for 10 min at 20 °C and the presence of phase-bright spores was checked under a phase-contrast microscope. Spore suspensions were stored at 4 °C until use.

### 4.3. Spore Structure Analysis

For transmission electron microscopy (TEM), freshly harvested and non-heat-treated spores were re-suspended in 40 mL of cold sterile water and washed four times by centrifugation at 1400× *g* for 20 min. Spores were prepared as previously described [45], except that post-fixation was performed with 1% osmium tetroxide. Four independent spore batches per sporulation temperature (45 °C and 55 °C) were used and up to 17 spores were observed per batch.

For scanning electron microscopy (SEM), freshly harvested and non-heat-treated spore suspensions were filtered on a 0.45-µm porosity membrane (Nucleopore, Whatman, Dutscher, Bernolsheim, France). Spores retained on the filter were fixed for 1 h at room temperature with 2.5% glutaraldehyde (*v*/*v*) in a 0.1 M sodium cacodylate buffer (pH 7.2) containing 1 mg/mL ruthenium red. The filter was washed three times in 0.2 M sodium cacodylate. Spores were post-fixed for 1 h at room temperature with 1% osmium tetroxide. Filters were then washed several times with water and placed in 30% ethanol. Spores were subjected to successive agitated dehydration baths containing increasing concentrations of ethanol (50%, 70%, 90%, and 100%). Next, samples were transferred into a hexamethyldisilazane (HMDS) bath until completely evaporated. Filters were pasted on a metallic support and gold plated. Observations were performed at 10 KV (SEM; Microscope FEI—Philips XL-30, Eindhoven, The Netherlands). Four batches were used per sporulation temperature and a total of up to 35 spores was observed per batch.

### 4.4. Spore Size Measurement

To measure the diameter of spores produced at 45 °C and 55 °C, SEM images were analyzed using ImageJ analysis software (v. 1.51j8, National Institutes of Health, Bethesda, MD, USA). A total of 73 and 78 spores from the four batches produced at 45 °C and 55 °C, respectively, was measured. The normality of the data distribution and the homogeneity of variance were tested using a Shapiro–Wilk test and a Fisher test, respectively. A Student’s *t*-test was performed to compare mean diameter values (Table S1). All statistical tests were conducted using XLSTAT (v. 2017.7) or utility software (Anastats Scop SARL, Rilly-sur-Vienne, France).

### 4.5. Spore Protein Extraction and Electrophoresis

Aliquots of 1 mL of spore suspensions (approx. 10^7^ spores/mL, depending on the batch) were centrifuged at 5000× *g* for 10 min at 4 °C. Spores were then resuspended in 1 mL of extraction buffer composed of 50 mM Tris-HCl buffer pH 7.5, 1 X protease-inhibiting cocktail (cOmplete^™^ mini Protease inhibitor cocktail EDTA-free Roche, Sigma-Aldrich, Saint-Quentin Fallavier, France) and 5 mM EDTA pH 8.0 (UltraPure^™^ 0.5 M EDTA, ThermoFisher Scientific, Villebon sur Yvette, France). Spores were lysed using 0.1-mm-diameter silica beads (MP biomedicals, Illkirch, France) in a Fast-Prep 120 machine (FastPrep^®^ FP120 Cell Disrupter, Thermo Savant, Illkirch, France). A series of 20 to 25 runs of 45 s at the maximal speed of 6.5 were performed until spores were completely lysed, as observed under a phase-contrast microscope. Samples were placed on ice for 1 min between runs to prevent protein degradation by over-heating.

Proteins were quantified using a colorimetric method based on the Bradford dye-binding assay, following the manufacturer’s instructions (Bio-Rad protein assay, Life Science, Marnes-la-Coquette, France). Extracted proteins were mixed with 1X Laemmli buffer (Bio-Rad, Life Science, Marnes-la-Coquette, France) to which 2.5% β-mercaptoethanol was added, and heated at 100 °C for 10 min. A short migration of the proteins was carried out on a 12% polyacrylamide gel using the following program: 20 min at 90 V and at 150 V to obtain a 5 mm in length migration. The gel was stained with imperial blue (Imperial^™^ Protein Stain, Thermo Fisher Scientific, Villebon sur Yvette, France) (Figure S3) and destained overnight in water; each lane was then cut in 3-mm squares for subsequent proteomic analysis.

### 4.6. LC-MS/MS Analysis

#### 4.6.1. Protein In-Gel Digestion

Each cut piece of gel was washed for 15 min with a 1:1 mixture of acetonitrile and 100 mM ammonium bicarbonate. The proteins contained in the gel were reduced with 10 mM dithiothreitol (Sigma, Saint Louis, MO, USA) and alkylated with 55 mM iodoacetamide (Sigma, Saint Louis, MO, USA) [63]. Digestion was performed in 50 mM ammonium bicarbonate (pH 8.0) and the quantity of modified trypsin (sequencing grade Promega, Madison, WI, USA) was adjusted in order to have an enzyme-to-protein ratio (*w*/*w*) equal to 1:50 in each sample. Digestion was carried out overnight at 37 °C and the supernatant was conserved. Peptides were extracted with 0.5% trifluoroacetic acid (TFA) in water/acetonitrile (*v*/*v*). Supernatants and extracted tryptic peptides were dried and resuspended at a concentration of 40 ng/µL in 0.08% (*v*/*v*) TFA and 2% (*v*/*v*) acetonitrile. We then injected 160 ng (according to the protein concentration measured prior to digestion) in the LC-MS/MS apparatus.

Peptide samples were solubilized in a buffer of 2% CH_3_CN, 0.08% TFA. Liquid chromatography was performed on a NanoLC Ultra system (Eksigent, AB SCIEX, Dublin, CA, USA). Samples were loaded at 7.5 μL min^−1^ on a C18 precolumn (5 μm, 100 μm i.d. × 2 cm length; NanoSeparations, Nieuwkoop, The Netherlands) connected to a separating Thermo PepMap 2 C18 column (2 μm, 75 μm i.d. × 25 cm length, Thermo Fisher Scientific, Waltham, MA, USA). Solvent A was 0.1% formic acid in water and solvent B was 0.1% formic acid in ACN. Peptide separation was achieved using a linear gradient from 5% to 30% of solvent B for 75 min at 300 nL min^−1^. Including the regeneration and the equilibration steps, a single run took 93 min. Eluted peptides were analyzed with a Q Exactive PLUS mass spectrometer (Thermo Fisher Scientific, San Jose, CA, USA) using a nanoelectrospray interface. Ionization was performed with a 1.3 kV spray voltage applied to an uncoated capillary probe (10μm i.d., New Objective, Littleton, MA, USA). Acquisition was performed in data-dependent mode and fragmentation occurred in HCD (Higher energy collisional dissociation). This included a full MS scan covering a mass-to-charge ratio (*m*/*z*) of 300 to 1400 with a resolution of 70,000 and an MS/MS step (normalized collision energy, 27%; resolution, 17,500). Dynamic exclusion was set to 40 s.

The mass spectrometry proteomics raw data were deposited in the PRIDE partner repository [64], as a ProteomeXchange dataset [65], with the identifier PXD029621 (http://www.ebi.ac.uk/pride/archive/projects/PXD029621, accessed on 8 November 2021).

#### 4.6.2. Identification and Quantification of Proteins 

Data were converted to mzXML format using MS convert (ProteoWizard v 3.0.8934). Proteins were identified using X!Tandem v.2015.04.01.1 [66] by matching peptides against the UniprotKB *Moorella thermoacetica* ATCC 39073 database containing 2452 entries (http://www.uni-prot.org/proteomes/UP000007053, accessed on 29 September 2021). Proteins were filtered and grouped using the X!TandemPipeline software v.3.4.2 [67], open-source software developed by PAPPSO (http://pappso.inrae.fr/bioinfo/xtandempipeline/, accessed on 29 September 2021). To eliminate the spectra created by the contaminants, data were also compared to a contaminant database.

Proteome identification was carried out with a precursor mass tolerance of 10 ppm and a fragment mass tolerance of 0.02 Da. Enzymatic cleavage rules were set to trypsin digestion (“after Arg and Lys, unless Pro follows directly after”) and no semi-enzymatic cleavage rules were allowed. The fix modification was set to cysteine carbamidomethylation and methionine oxidation was considered as a potential modification. In a second pass, N-terminal acetylation was added as another potential modification, but all other previous settings were retained. The identified proteins were filtered as follows: (1) peptide E-value < 0.05 with a minimum of 2 peptides per protein; and (2) a protein E-value of <10^−4^. Table S3 contains information on the number of spectra of at least 2, in at least one condition, for the 45 °C and the 55 °C samples and FDR peptides = 0.02% and FDR proteins = 0.09%. Table S4 reports peptides corresponding to proteins identified in Table S3.

Protein abundance values were calculated using spectral counts from three independent biological replicates. We used a rough semi-quantitative method, which allows us to detect large variations in abundance, including presence/absence variations [68,69,70]. In addition, normalization was done according to the method

(1) $$n\times \left(\frac{Ms}{Ts}\right)$$

where *n* refers to the number of spectra identified per protein in the sample, *Ms* refers to the mean sum of spectra identified for each sample, and *Ts* refers to the total number of spectra per sample.

MassChroqR (version 0.3.7), an R toolbox developed by the PAPPSO platform (http://pappso.inrae.fr/, accessed on 29 September 2021), was used to check the quality of the data and perform statistical analysis on the proteomic data. The significance of variation was determined by an ANOVA (analysis of variance) test in all analyses. The obtained *p*-values were adjusted for multiple testing by the Benjamini–Hochberg approach [71]. The padjust_values obtained from the ANOVA for the proteomic data were considered significant below a value of 0.05 in SC.

### 4.7. Identification of Spore-Associated Proteins

Using the Blast-P tool (https://blast.ncbi.nlm.nih.gov/Blast.cgi, accessed on 29 September 2021), the amino-acid sequences of the spore-associated proteins from several spore-forming species [34,35,37,38,72] were used to query the *M. thermoacetica* ATCC 39073 genome. To define a “hit”, we considered several criteria: % of identity or homology, % of protein coverage, and a minimal E-value of 2.00 × 10^−6^.

## Figures and Tables

**Table 1:** Spore-related proteins identified in *Moorella thermoacetica* ATCC 39073.

*Moorella thermoacetica* ATCC 39073 LocusS ^a^	Protein ID ^b^	*M. thermoacetica* Protein Identification ^b^	Putative Protein Name ^c^	Hypothetical Function ^d^	Putative Localization ^d^	ProteinsS of Bs/Bc/Ba/Cd/Cp ^c,d^
moth_1518	Q2RIB2	Ferredoxin-NADP(+) reductase subunit alpha	-	Nd ^e^	Exosporium	CD1536
moth_1286 moth_1879	Q2RIY9 Q2RHB0	Rubrerythrin	Rbr	Likely to play a role in spore resistance	Exosporium	CD0825 CD1524
moth_0602	Q2RKV7	Uncharacterized protein	-	nd	Exosporium	CD2434
moth_1747 moth_1815	Q2RHP1 Q2RHH4	Uncharacterized protein/Agmatinase	Arginase	nd	Exosporium	BAS0155 BAS2260
moth_2167	Q2RGI3	Alanine racemase	Alr	Spore protection	Exosporium	BSU17640 BAS0238
moth_1405	Q2RIM1	Nucleoside recognition	FeoB	nd	Exosporium	CD1517
moth_1683	Q2RHV4	Cation diffusion facilitator family transporter	-	nd	Exosporium	CD0902
moth_0034	Q2RMG5	2-oxoglutarate synthase	-	nd	Exosporium	CD0117
moth_0033	Q2RMG6	Pyruvate flavodoxin/ferredoxin oxidoreductase-like protein	-	nd	Exosporium	CD0116
moth_0035	Q2RMG4	2-oxoacid: acceptor oxidoreductase, gamma subunit, pyruvate/2-ketoisovalerate	-	nd	Exosporium	CD0118
moth_0266	Q2RLT8	Enolase	Eno	nd	Coat/exosporium	CD3170
moth_0837	Q2RK85	Stage V sporulation protein D	SpoVD	mother-cell specific penicillin-binding protein (spore cortex)	Coat/exosporium	BC3915
moth_2301	Q2RG51	Kynurenine formamidase	-	Metal-dependent hydrolase	Coat/exosporium	BC0395
moth_0739	Q2RKI2	Polysaccharide deacetylase	YlxY or Pda or PdaB	PdaA/PdaB:spore cortex peptidoglycan synthesis	Coat/exosporium	CD2598
moth_1414	Y1414_MOOTA	UPF0597 protein moth_1414	-	nd	Coat/exosporium	CD630
moth_0738	Q2RKI3	Peptidase M1, membrane alanine aminopeptidase	-	nd	Coat/exosporium	CD3652
moth_1693	Q2RHU4	Protein translocase subunit YajC	YajC	nd	Coat/exosporium	BC4410
moth_1319	Q2RIV7	Stage IV sporulation protein A	SpoIVA	Anchors the spore coat to the spore surface via SpoVM	Spore coat basement	BSU22800 BC1509 CD2629
moth_1782 ^f^	Q2RHK6	CoatF	-	nd	Coat	-
moth_1059	Q2RJL6	Peptidase M16-like protein	YmxG	nd	Coat	BC3786
moth_1391	Q2RIN5	Spore coat peptide assembly protein CotJB	CotJB	nd	Inner coat	BSU06900 BC0822 CD630
moth_1392	Q2RIN4	Manganese containing catalase	CotJC	May protect against oxydative stress	Inner coat	BSU06910 BC0821 CD0598 CD2401 CPR0934
moth_0257	Q2RLU7	HAD-superfamily hydrolase subfamily IIB	YhaX	Protection of the spore	Spore coat basement	BSU09830
moth_1069	Q2RJK6	Ribonuclease J	RnjA	RNA processing	Coat	BC3977
moth_2016	Q2RGX7	CoatF-like protein	YhcQ	nd	Coat	BSU30910
moth_1365	Q2RIR1	Coat protein SA	CotSA	Spore resistance	Coat	BSU30910
moth_1126	Q2RJE9	Amino acid ABC transporter substrate-binding protein, PAAT family	TcyA (YckK)	Cystine uptake	Coat	BSU03610
moth_1016	Q2RJQ9	Spore coat protein manganese catalase	CotG	nd	Coat	CD1567
moth_1693	Q2RHU4	Protein translocase subunit YajC	YajC/YrbF	nd	Inner membrane/coat	BSU27700
moth_1916	Q2RH73	Superoxide dismutase	SodF/SodA	Detoxication of oxygen radicals	Coat	BSU19330 BC1468 CD1631
moth_0056	Q2RME4	Sporulation-specific protease	YabG	Modification of spore coat proteins	Coat	BSU00430 BC0047 CD3569 CPR2191
moth_0373	Q2RLI2	Putative transcriptional regulator	YkvN	MarR/DUF24 family transcription regulator	Coat	BSU13760
moth_0426	Q2RLD2	Short-chain dehydrogenase/reductase	YhxC	Similar to alcohol dehydrogenase	Coat	BSU10400
moth_0517	Q2RL42	N-acetylmuramoyl-L-alanine amidase	-	Involved in germination	Coat	BC2207
moth_0527	Q2RL32	Trigger Factor	-	nd	Coat	BC4480
moth_0063	Q2RMD7	Glycoside hydrolase, family 18	YaaH ot YdhD	YaaH: spore germination cortex lytic enzyme YdhD: spore coat peptidoglycan hydrolase	Inner coat (YaaH) Coat (YdhD)	BSU01160/BSU05710 BC3607
moth_0088	Q2RMB2	Uncharacterized protein	YabP	Required for sporulation at a late stage	Outer membrane	BSU00600 BC0063 CPR2486
moth_1357 ^g^ moth_1058	Q2RIR9 Q2RJL7	Serine-type D-Ala-D-Ala carboxypeptidase	DacB	Sporulation-specific carboxypeptidase involved in spore cortex peptidoglycane cross-linking	Cortex	BSU23190 CPR1770
moth_0201	Q2RM03	Propeptide, PepSY amd peptidase M4	YpeB	Germination protein, essential for SleB assembly in spores	Inner membrane	BSU22920 BC2752
moth_1499	Q2RID1	Serine-type D-Ala-D-Ala carboxypeptidase	DacF	Penicillin-binding protein I	Inner membrane	BSU23480 BC4075 CD1291 CPR1775
moth_0887	Q2RK35	ATPase, E1-E2 type	AtcL	Similar to the *E. coli* magnesium transporter	Inner membrane	BSU15650
moth_0734 moth_0054 moth_0202	Q2RKI7 Q2RME6 Q2RM02	Cell wall hydrolase	SleB	Spore cortex-lytic enzyme involved in germination	Outer surface of the inner spore membrane	BSU13930 BC2753
moth_1358	Q2RIR8	Uncharacterized protein	GerW/YtfJ	Germination protein	Inner membrane	BSU29500 BC4640/BC2095
moth_0926	Q2RJZ6	Germination protease	Gpr	Degradation of SASPs	Inner membrane/core	BSU25540 BC4319 CPR2013
moth_2417 moth_0736	Q2RFU0 Q2RKI5	Peptidase S1 and S6, chymotrypsin/Hap	YyxA	Similar to quality control membrane serine protease HtrA	Inner membrane	BSU40360
moth_0925	Q2RJZ7	Small acid-soluble spore protein, alpha/beta type	SspA	Protection of spore DNA	Core	BSU29750 CD2688
moth_0806	Q2RKB5	Small, acid-soluble spore protein, alpha/beta family	SspF	Protection of the spore DNA	Core	BSU24210
moth_1875	Q2RHB4	Small, acid-soluble spore protein	SASP	Small, acid-soluble spore protein	Core	CPR1870
moth_2056	Q2RGT7	NADH:flavin oxidoreductase/NADH oxidase	YqiG	nd	nd	BSU24210
moth_1272	Q2RJ03	N-acetylmuramoyl-L-alanine amidase	CwlC	Sporulation-specific N-acetylmuramoyl_L-alanine amidase	nd	BSU17410
moth_1828	Q2RHG1	Uncharacterized protein	YckD	nd	nd	BSU03400
moth_1059	Q2RJL6	Peptidase M16-like protein	YmxG	Control of proteolytic activity	nd	BSU16710
moth_1356	Q2RIS0	Nucleoside recognition	SpmA	Spore maturation protein, spore core dehydration, involved in germination	nd	BSU23180 BC1470
moth_1355	Q2RIS1	Nucleoside recognition	SpmB	Spore maturation protein, spore core dehydration, involved in germination	nd	BSU23170 BC1471
moth_1064	Q2RJL1	Alanine dehydrogenase/PNT-like protein	SpoVFA	Dipicolinate synthase (subunit A)	nd	BSU16730 BC3801
moth_1065	Q2RJL0	Flavoprotein	SpoVFB	Dipicolinate synthase (subunit B)	nd	BSU16740 BC3800

^a^*Moorella thermoacetica* ATCC 39073 database [28]; ^b^ Information from the UniProtKB database (http://www.uniprot.org, accessed on 29 September 2021); ^c^ All proteins were identified by Blast-P from the spore coat and/or exosporium of *Bacillus subtilis* 168 (Bs, BSU), *B. cereus* ATCC 14579 (Bc, BC), *B. anthracis* Sterne (Ba, BAS), *C. difficile* 630 (Cd, CD), and *C. perfringens* (Cp, CPR); ^d^ Data obtained from Abhyankar et al., 2011 [34]; Abhyankar et al., 2013 [35]; Paredes-Sabja et al., 2014 [36]; Diaz-Gonzalez et al., 2015 [37]; Stewart, 2015 [38]; Abhyankar et al., 2016 [23]; Subtiwiki (http://subtiwiki.uni-goettingen.de/, accessed on 29 September 2021); ^e^ nd: protein function and/or localization not determined; ^f^ Protein annotated as Coat F protein in the *M. thermoacetica* genome but with spore coat proteins of *B. subtilis*; ^g^ Amino acid sequences encoded by *moth_1058* and *moth_1357* are highly similar. The *dacB* gene is described as being in an operon with the *spmA* and *spmB* genes. Thus, the *moth_1357* locus more likely encodes DacB.

**Table 2:** Spore-associated proteins identified by proteomic analysis of *Moorella thermoacetica* ATCC 39073 spores produced at 55 °C and 45 °C.

Protein ID ^a^	*Moorella thermoacetica* Protein Identification ^a^	Mean Spectral Counts		*p* Adjust Value
		55 °C ^b^	45 °C ^b^	
Q2RIR1	Glycosyl transferase (CotSA)	**5.3 ± 2.9 **	**0.0 ± 0.0 **	**0.0002 **
Q2RIV7	Stage IV sporulation protein A (SpoIVA)	**41.0 ± 6.6 **	**21.3 ± 11.5 **	**0.0007 **
Q2RGT7	NADH:flavin oxidoreductase/NADH oxidase (YqiG)	**11.3 ± 7.6 ^c^**	**2.7 ± 3.1 **	**0.0016 **
Q2RIB2	FAD/NAD(P)-binding oxidoreductase	**53.0 ± 4.6 **	**32.0 ± 4.6 **	**0.0027 **
Q2RHK6	Coat protein F ^d^	**9.7 ± 3.2 **	**2.3 ± 2.1 **	**0.0045 **
Q2RM03	Propeptide, PepSY amd peptidase M4 (YpeB)	**3.3 ± 3.1 ^e^**	**0.0 ± 0.0 **	**0.0057 **
Q2RJL6	Peptidase M16-like protein (YmxG)	**8.3 ± 1.2 **	**2.3 ± 0.6 **	**0.0190 **
Q2RH73	Superoxide dismutase (SodF)	**2.3 ± 2.1 ^e^**	**0.0 ± 0.0 **	**0.0275 **
Q2RIN4	Manganese containing catalase (CotJC)	**8.3 ±± 4.7 **	**1.7 ± 1.5 **	**0.0043 **
Q2RGX7	Coat protein YhcQ	6.0 ± 1.7	2.0 ± 2.0	0.0949
Q2RIY9	Rubrerythrin (Rbr)	30.0 ± 4.5	41.0 ± 3.0	0.1141
Q2RMG5	2-oxoglutarate synthase	1.0 ± 1.0	0.0 ± 0.0	0.1841
Q2RGI3	Alanine racemase (Alr)	8.0 ± 1.0	4.0 ± 1.0	0.1907
Q2RJQ9	Catalase (CotG)	2.3 ± 0.6	0.7 ± 1.2	0.2825
Q2RJE9	Amino acid ABC transporter substrate-binding protein (TcyA/YckK)	4.0 ± 1.0	1.7 ± 1.5	0.2825
Q2RHU4	Protein translocase subunit (YajC)	0.7 ±0.6	2.3 ± 2.3	0.2825
Q2RIN5	Spore coat protein (CotJB)	0.7 ± 1.2	0.0 ± 0.0	0.2931
Q2RJZ7	Small acid-soluble spore protein, alpha/beta type (SspA)	5.0 ± 1.0	2.7 ± 1.2	0.3763
Q2RLT8	Enolase (Eno)	46.0 ± 6.5	53.0 ± 7.2	0.4941
Q2RHH4	Agmatinase	1.3 ± 1.2	2.7 ± 1.2	0.5072
Q2RKV7	Hypothetical protein	2.7 ± 1.2	3.3 ± 1.5	0.8454
Q2RLU7	HAD-superfamily hydrolase subfamily IIB (YhaX)	5.7 ± 1.2	6.3 ± 1.2	0.8787
Q2RHB0	Rubrerythrin (Rbr)	20.0 ± 3.2	21.3 ± 2.3	0.9068
Q2RJK6	Ribonuclease J (RnjA)	3.0 ± 0.0	3.3 ± 0.6	0.9207

^a^ Information from the UniProtKB database; ^b^ Spectral counting performed on three independent biological replicates; proteins whose abundance varies significantly between 45 °C and 55 °C are in bold (*p* adjust_value < 0.05) (*n* = 3); ^c^ Overlap of standard deviations; ^d^ Protein annotated as CoatF protein in the *M. thermoacetica* genome not homologous with the spore coat proteins of *B. subtilis*; ^e^ No spectra found in one of the three biological replicates.

## Data Availability

The raw LC-MS/MS data were deposited in the PRIDE partner repository as a ProteomeXchange dataset (PXD029621).

## Supplementary Materials

The following supporting information can be downloaded at: https://www.mdpi.com/article/10.3390/ijms23010550/s1.
