# Supplementary material for: Insights into the Structure and Protein Composition of Moorella thermoacetica Spores Formed at Different Temperatures
Source: Int J Mol Sci. 2022 Jan 4;23(1):550. doi: 10.3390/ijms23010550 (PMC8745062; doi:10.3390/ijms23010550)
Supplement: Supplementary file 1 [file ijms-23-00550-s001.zip › Figures.pdf]

Supplementary Material

Insights into the Structure and Protein Composition of *Moorella thermoacetica* Spores Formed at Different Temperatures

Tiffany Malleck [1,2], Fatima Fekraoui [2], Isabelle Bornard [3], Céline Henry [4], Eloi Haudebourg [4], Stella Planchon [2,*] and Véronique Broussolle [1,*]

[1] INRAE, Avignon Université, UMR SQPOV, F-84000 Avignon, France
[2] Unité EMaiRIT'S, CTCPA, F-84911 Avignon, France
[3] INRAE, Pathologie végétale, F-84143 Montfavet, France
[4] PAPPSO, Micalis Institute, INRAE, AgroParisTech, Université Paris-Saclay, F-78352 Jouy-en-Josas, France
* Correspondence: veronique.broussolle@inrae.fr (V.B.); splanchon@ctcpa.org (S.P.); Tel.: +33-(0)432722518 (V.B.); +33-(0)490843246 (S.P.)

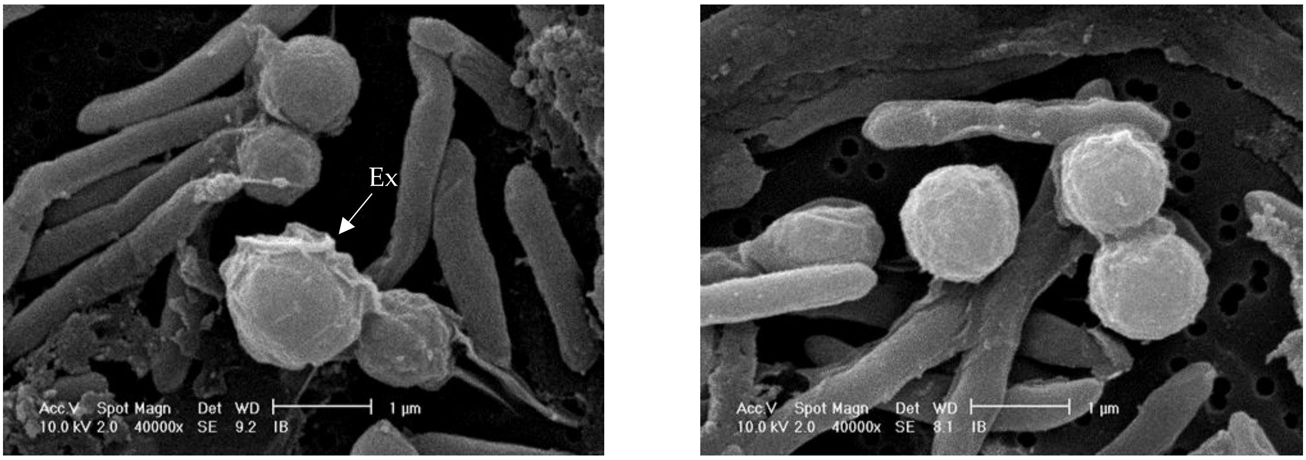

Figure S1. Spores of *Moorella thermoacetica* strain ATCC 39073, produced on agar plates at 45 °C and observed by scanning electron microscopy. The exosporium (Ex) is indicated by the white arrow.

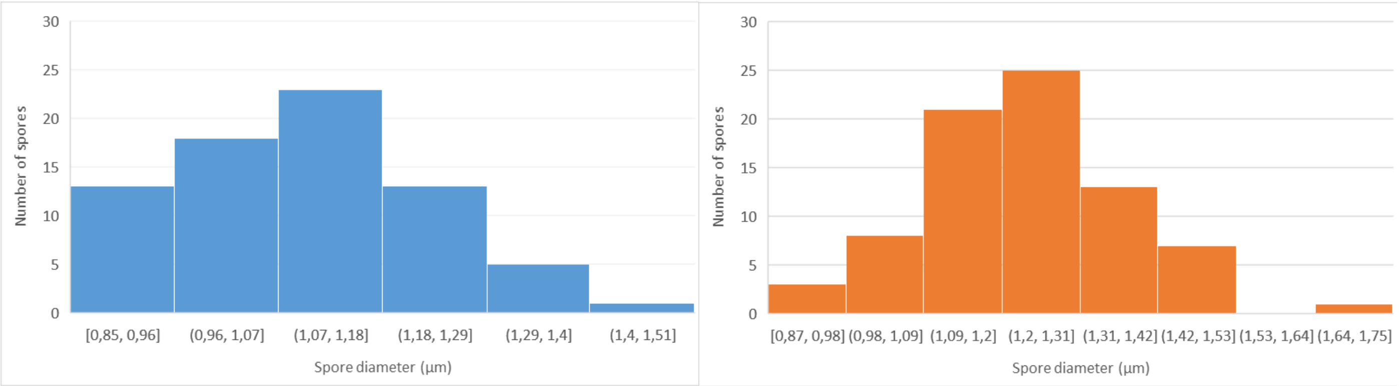

Figure S2. Heterogeneity of spore diameters of *Moorella thermoacetica* at 45 °C (blue bars) and 55 °C (orange bars), measured on SEM images.

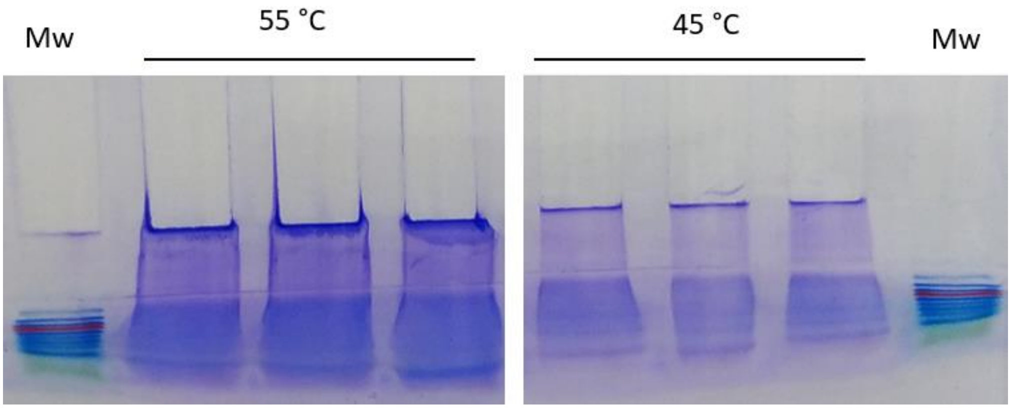

Figure S3. SDS-PAGE gel (short migration) of *Moorella thermoacetica* spore protein extracts (three biological replicates for 45 °C and for 55 °C) used for LC-MS/MS analysis. Mw, molecular weight ladder.
